# Impact of Saturated and Unsaturated Oils on the Nonlinear Viscoelasticity, Microstructure, and 3D Printability of Fish Myofibrillar-Protein-Based Pastes and Gels

**DOI:** 10.3390/gels11040295

**Published:** 2025-04-16

**Authors:** Timilehin Martins Oyinloye, Won Byong Yoon

**Affiliations:** 1 Department of Food Science and Biotechnology, College of Agriculture and Life Sciences, Kangwon National University, Chuncheon 24341, Republic of Korea; oyinloyetm@kangwon.ac.kr; 2 Elder-Friendly Food Research Center, Agriculture and Life Science Research Institute, Kangwon National University, Chuncheon 24341, Republic of Korea

**Keywords:** fish myofibrillar protein, nonlinear rheology, SAOS, LAOS, gel microstructure, oil incorporation, 3D printing

## Abstract

The effect of oil incorporation (soybean oil [SO] and coconut oil [CO] at 0, 1, 3, and 5 g/100 g) on the rheological, structural, and 3D printing properties of fish myofibrillar protein (MP, also known as surimi) paste and gel was investigated. Small-amplitude oscillatory shear (SAOS) tests showed that increasing oil concentration reduced the storage modulus (G′), weakening the gel network. Large-amplitude oscillatory shear (LAOS) analysis revealed strain-stiffening shifts and nonlinearity at γ = 5%. CO-containing gels exhibited higher hardness and gumminess, particularly at lower concentrations, due to enhanced protein–lipid interactions. In contrast, SO-containing gels showed reduced strength at higher concentrations, indicating phase separation. SEM confirmed that CO promoted a denser network, while SO led to a more porous structure, especially at 5% oil. Three-dimensional printing analysis demonstrated that both oils improved extrusion flowability by reducing nozzle friction. However, CO-containing samples maintained post-extrusion stability at 85% moisture, whereas SO-containing samples collapsed after multiple layers due to excessive softening. These findings highlight oil’s dual role in MP gels, enhancing lubrication and flowability while compromising rigidity. The results offer valuable insights for developing soft, texture-controlled foods using 3D printing, especially for personalized nutrition applications such as elderly care or dysphagia-friendly diets.

## 1. Introduction

Myofibrillar proteins (MPs) are the primary structural proteins in fish muscle, composed predominantly of myosin, actin, tropomyosin, and troponin [1]. These proteins are essential for the textural and functional attributes of processed meat and seafood products, such as surimi-based seafood products, due to their ability to form heat-induced gels. In the paste state, MP exhibits weak gel-like behavior, characterized by moderate viscosity and yield stress, which enables flow under applied stress [2]. Upon heating, MP undergoes thermal denaturation and aggregation, forming a three-dimensional protein network responsible for the gel structure and mechanical properties of processed products [3].

While MP or surimi gelation has been extensively studied, the influence of lipid incorporation on its structural and rheological properties, particularly in the context of advanced food structuring methods like 3D printing, remains an area of emerging interest. Oil incorporation in MP-based gels can significantly modify their physicochemical properties, influencing water retention, gel strength, and texture [4]. The type and concentration of oil play a crucial role in determining the stability and distribution of the lipid phase within the protein matrix. Saturated fats, such as coconut oil (CO), with their higher solid fat content at room temperature, may enhance the rigidity of MP gels through increased protein–lipid interactions. Conversely, unsaturated oils like soybean oil (SO), rich in polyunsaturated fatty acids (PUFAs), may improve the lubrication properties and modify gel network formation by interfering with protein–protein interactions [5]. Understanding these effects is crucial for optimizing the flowability and structural integrity of MP-based pastes during industrial processing, where factors such as extrusion, molding, and 3D printing require precise control over rheological behavior.

Several studies have investigated the effects of lipid incorporation on MP gels. Wu et al. [6] investigated MP–olive oil composite gels, finding that the type of emulsifier used influenced the gel’s rheology and microstructure. Their results indicated that non-meat proteins as emulsifiers could modify the textural properties of MP gels. Similarly, Wu et al. [7] studied the impact of emulsion droplet size and membrane type on MP–plant lipid composite gels, concluding that smaller oil droplets and protein-coated droplets enhanced the gel’s rheological properties and stability. Additionally, recent research has explored the role of low-molecular-weight additives, including edible oils, in MP gelation, highlighting how these additives modify intermolecular forces and influence the final gel properties [8,9]. Collectively, these findings emphasize the importance of understanding both linear and nonlinear viscoelastic properties and microstructural characteristics to optimize MP gels for various food processing applications.

Rheological characterization is essential in evaluating the processability of MP pastes and gels. Traditional small-amplitude oscillatory shear (SAOS) testing provides insights into viscoelastic properties under low-strain conditions. However, real-world food processing operations often involve large deformations, necessitating nonlinear rheological analysis [10]. Large-amplitude oscillatory shear (LAOS) testing enables a more comprehensive understanding of the strain-dependent behavior of MP gels, capturing changes in elasticity, structural resilience, and energy dissipation under industrially relevant conditions [2]. Ewoldt et al. [9] introduced a framework with Chebyshev polynomial decomposition to analyze nonlinear viscoelasticity, offering a more nuanced interpretation of LAOS data. This methodology has been applied to various complex fluids, providing deeper insights into their nonlinear behaviors. Given the increasing interest in MP-based products for novel food applications, a systematic investigation into their nonlinear viscoelastic properties in the presence of oil is warranted.

While studies have explored the nonlinear rheological properties of MP gels, limited research has focused on the combined effects of oil incorporation and nonlinear viscoelastic behavior. Oyinloye and Yoon [2] investigated the impact of moisture content on the nonlinear viscoelastic behavior of fish MP pastes, demonstrating the significance of moisture levels on structural integrity and processability. Similarly, Liu et al. [11] examined how pH variations influence the linear and nonlinear viscoelastic properties of tuna MP, showing that pH adjustments markedly affect gel network formation and stability. However, comprehensive analyses integrating oil incorporation with nonlinear rheological assessments, particularly in the context of advanced food structuring techniques like 3D printing, remain scarce. This highlights the need for studies that concurrently evaluate the influence of lipid addition on both nonlinear viscoelastic properties and the functional performance of MP-based systems.

The advent of 3D printing in food manufacturing introduces additional complexity, as successful printing depends on achieving a balance between printability, extrusion flow behavior, and post-deposition stability. MP pastes must possess sufficient viscosity to ensure uniform extrusion while maintaining structural integrity before subsequent heat-induced gelation [12]. The inclusion of oil is expected to influence these factors by modifying viscosity, gelation kinetics, and interlayer adhesion. However, the extent to which different oil types and concentrations impact printability and post-extrusion structural retention remains underexplored.

Beyond rheological and mechanical considerations, the microstructural characteristics of MP gels play a critical role in determining their overall functionality. Scanning electron microscopy (SEM) provides valuable insights into protein–lipid interactions at the microscopic level, revealing changes in network porosity, phase separation, and oil distribution [4,13]. Previous studies have shown that oil incorporation can either reinforce or disrupt the protein matrix depending on lipid composition and phase behavior [14]. By combining microstructural analysis with rheological and mechanical assessments, this study aims to establish a comprehensive understanding of how oil incorporation affects MP gel properties.

Therefore, this study aims to characterize the effects of incorporating saturated and unsaturated oils on the rheological and microstructural properties of fish myofibrillar protein (MP) paste and gel. Specifically, this study investigates how oil type (coconut oil and soybean oil) and concentration influence gelation behavior and nonlinear viscoelastic responses, using large-amplitude oscillatory shear (LAOS) rheology. Additionally, the study evaluates the 3D printability and post-extrusion stability of MP pastes to determine their suitability for structured food manufacturing. The scientific hypothesis is that the type and concentration of oil will distinctly modulate the viscoelastic properties, microstructure, and mechanical strength of MP-based gels, which, in turn, will influence their flow behavior and 3D printability. Saturated fats like CO are expected to reinforce the gel network and enhance structural stability, even under high-moisture conditions, due to increased solid fat content and protein–lipid interactions. In contrast, unsaturated oils like SO may improve lubrication and flow during extrusion but potentially reduce structural retention after deposition.

The results from this research may be utilized within the food industry for designing customized, soft-textured protein-based foods using 3D printing technology, especially for healthcare and aging populations requiring modified texture diets. Furthermore, the findings provide insights into oil–protein interactions that can be used to improve the formulation of surimi-based and other structured protein products.

## 2. Results and Discussion

### 2.1. Linear Rheological Behavior of MP Paste and Gel

#### 2.1.1. Strain Sweep Analysis of MP Paste and Gel

**Figure 1:**
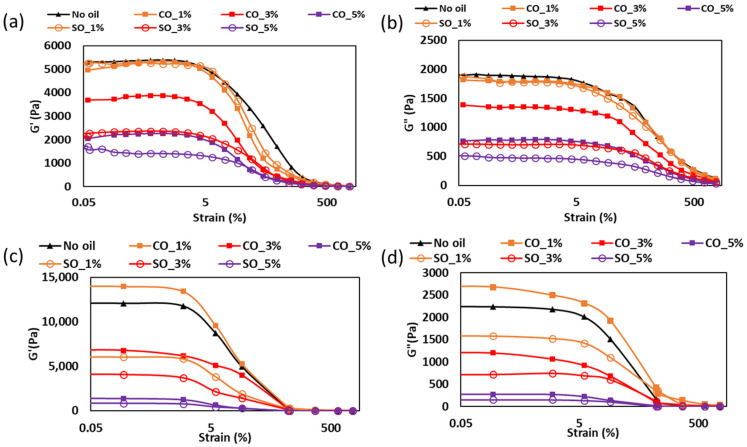
Strain sweep analysis for MP with different oil content obtained at frequency 1 Hz: (**a**) storage modulus for the paste, (**b**) loss modulus for paste, (**c**) storage modulus for gel, and (**d**) loss modulus for gel.

The strain-dependent viscoelastic properties of MP paste and gel with different concentrations of coconut oil (CO) and soybean oil (SO) conducted at 20 °C are illustrated in Figure 1. At this testing temperature, the molecular interactions between protein and oil, influenced by the physical state and chemical composition of the oils, played a crucial role in determining the structural stability and mechanical strength of the samples. In the paste state (Figure 1a,b), the samples without oil, as well as those with 1% CO and SO, exhibited similar G′ and G″ values within the LVR (0–10% strain). However, at higher oil concentrations, both moduli decreased, with a more pronounced reduction in samples containing soybean oil. This difference may result from variations in the chemical structure of the oils, particularly their degree of saturation. Coconut oil, rich in saturated medium-chain fatty acids such as lauric acid (C12:0) and myristic acid (C14:0), is known to have stronger hydrophobic interactions with proteins [15]. At 20 °C, after paste preparation, CO was in a semi-solid state, which could have contributed to partial reinforcement of the paste structure by restricting molecular mobility within the protein network. In contrast, soybean oil, composed primarily of unsaturated fatty acids, particularly linoleic acid (C18:2) and oleic acid (C18:1), remained liquid at 20 °C. This liquid nature may have interfered with protein–protein interactions, increasing molecular fluidity and resulting in a weaker paste structure.

The decrease in viscoelastic properties with increasing oil concentration suggests that excessive oil disrupted protein–protein interactions within the paste. Kim et al. [16] found that MP pastes containing unsaturated oils exhibited weaker viscoelasticity due to phase separation, leading to a less cohesive protein network. Similarly, Chen et al. [17] demonstrated that liquid oil in protein matrices reduced intermolecular stabilization, making the paste less resistant to deformation.

The differences in G′ and G″ values between CO and SO samples could also be linked to the potential for chemical interactions between oil molecules and protein structures. Saturated fatty acids in CO have been reported to promote stronger hydrophobic interactions with MP myosin, which may potentially aid in the formation of localized protein aggregates that stabilized the paste network [4]. In contrast, the higher proportion of unsaturated fatty acids in SO could lead to increased protein–lipid interactions via electrostatic and van der Waals forces, which are generally weaker than hydrophobic interactions and may have resulted in a more fluid-like paste consistency.

In the gel state (Figure 1c,d), a different trend was observed; the sample containing 1% CO exhibited the highest G′ and G″ values, followed by the No oil sample, with increasing oil concentrations leading to a progressive reduction in viscoelasticity. This suggests that oil interacted with the myosin gelation process during thermal treatment, potentially influencing protein denaturation and network formation. During heat-induced gelation, myosin undergoes conformational changes that expose reactive hydrophobic and sulfhydryl (-SH) groups, facilitating intermolecular crosslinking and gel stabilization [18]. The presence of oil could influence this process in several ways. CO, being liquid during the heating process and composed of medium-chain saturated fatty acids, may have been partially entrapped within the protein matrix, and, upon cooling, forming a semi-solid state at 20 °C may stabilize the gel network by limiting excessive molecular mobility. This aligns with the findings of Zhou et al. [4], who reported that the incorporation of saturated fatty acids into fish MP gels resulted in stronger viscoelastic properties due to enhanced hydrophobic interactions.

On the other hand, SO remained in a liquid state throughout the entire gelation and cooling process. The continuous phase of liquid oil in SO-containing gels may have disrupted protein–protein crosslinking, reducing the formation of a strong myosin network. Zhang et al. [19] found that myofibrillar protein containing high levels of unsaturated oil exhibited lower viscoelasticity due to limited disulfide bond formation, which is essential for structural stability. The reduction in G′ and G″ observed in SO samples in this study aligns with such findings, indicating that liquid oil impeded efficient gel formation especially at a concentration >3%.

Furthermore, it is possible that some of the oil molecules were incorporated into the protein network rather than merely coexisting as separate phases. Research by Xu et al. [20] indicates that protein–lipid interactions during gelation can lead to the partial entrapment of lipids within the protein matrix, particularly when the lipid phase is well dispersed. The stronger G′ observed in CO samples suggests that CO contributed more positively to the gel’s viscoelastic response, possibly by influencing the protein network’s organization or restricting molecular mobility during cooling rather than merely coexisting as a separate phase. In contrast, the weaker gels in SO samples imply that liquid oil may have disrupted protein crosslinking rather than being fully incorporated into the network.

Compared to plant-based protein systems, such as those formed with soy or pea protein isolates, the MP system in this study shows distinct behavior. In soy and pea protein gels, oil addition, especially of unsaturated oils, typically reduces gel strength due to the disruption of protein–protein interactions [21]. However, unlike MP, plant-based systems lack myosin and associated heat-induced gelation mechanisms, relying instead on globulin aggregation and disulfide bonding. These systems often require additional crosslinkers or polysaccharides to achieve comparable mechanical strength [22]. In particular, Ningtyas et al. [21] showed that adding soybean oil to soy protein isolate gels weakened their structure, a trend that is consistent with our observations in the MP system. However, the ability of saturated oils like CO to reinforce the MP gel matrix is less evident in plant proteins, possibly due to a lower affinity between saturated fatty acids and plant protein structures. Therefore, the advantage of MP systems lies in their inherent gelation capacity and stronger response to hydrophobic interactions with saturated fats, enabling better control over texture and structural integrity. This highlights the importance of considering both oil composition and protein system when formulating protein-based food products for optimized textural and functional properties.

#### 2.1.2. Frequency Sweep Analysis of MP Paste and Gel

**Figure 2:**
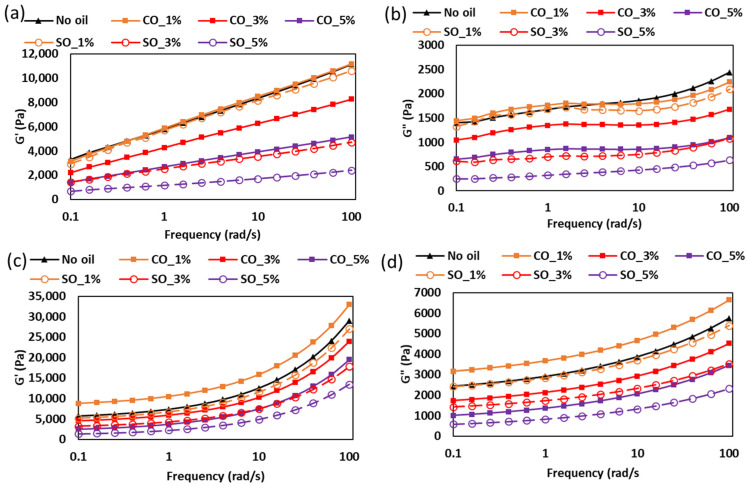
Rheological properties of MP paste and gel with different oil type and concentrations: (**a**) storage modulus for the paste, (**b**) loss modulus for paste, (**c**) storage modulus for gel, and (**d**) loss modulus for gel.

The frequency-dependent viscoelastic properties of MP paste and gel with different concentrations of CO and SO are shown in Figure 2. This analysis provides insights into the structural stability and mechanical response of the protein network under dynamic conditions, which is critical for understanding the behavior of these systems in various food applications. In the paste state (Figure 2a,b), both G′ and G″ increased with frequency across all samples, indicative of a weak gel-like structure where elastic properties dominate at higher deformation rates. At lower frequencies (1–10 rad/s), samples without oil (No oil), with 1% CO, and with 1% SO exhibited similar G′ and G″ values, suggesting that minimal oil addition does not significantly disrupt the protein network at lower shear. This behavior aligns with the findings of Zhou et al. [4], who demonstrated that low lipid concentrations (<2%) can integrate into the protein matrix without disrupting critical junction zones. However, the frequency-dependent divergence, particularly the marked reduction in SO_1% moduli at >10 rad/s, indicates fundamental differences in how these oils interact with the protein network under deformation.

CO’s superior performance at higher frequencies stems from its saturated fatty acid composition (primarily lauric and myristic acids), which promotes stronger hydrophobic interactions with nonpolar protein residues. Spectroscopic studies indicate that saturated medium-chain triglycerides preferentially bind to hydrophobic regions of myosin, reinforcing the protein scaffold [23,24]. In contrast, soybean oil’s polyunsaturated fatty acids (linoleic and linolenic acids) create more fluid interfaces that weaken hydrophobic associations under shear, as evidenced by the reduced moduli at elevated frequencies [19].

The concentration-dependent modulus reduction observed for both oil types suggests the presence of competing interfacial phenomena. At higher concentrations (3–5%), oil droplets likely exceed the protein’s emulsification capacity, leading to three primary effects: protein network dilution, formation of discontinuous oil domains, and increased interfacial slippage [25,26]. Chojnicka-Paszun et al. [27] studied the lubrication effect of fat content in milk and demonstrated that excess oil creates lubricated interfaces between protein aggregates, facilitating relative motion under shear. This lubrication effect becomes particularly pronounced at higher frequencies where the Deborah number approaches unity, explaining the accelerated modulus reduction observed in our study. The stronger reduction effect in SO-containing samples may reflect its lower interfacial tension with the aqueous protein phase compared to CO [28].

Upon thermal gelation (Figure 2c,d), the viscoelastic properties of the samples exhibited notable changes. The CO_1% gel demonstrated significantly higher G′ and G″ values compared to other samples, indicating enhanced gel strength. This enhancement can be attributed to the semi-solid nature of CO below room temperature (<27 °C), which, when integrated into the protein matrix during gelation, may act as a filler, restricting molecular mobility and promoting a denser network. In addition, the superior performance of CO_1% gels suggests that thermal treatment enhances hydrophobic interactions between saturated lipids and protein aggregates. The comparative study on fat types conducted by Sulaiman et al. [29] showed that saturated fats increase the thermal stability of myofibrillar networks compared to unsaturated fats. This stabilization effect explains CO’s ability to maintain higher moduli across the frequency spectrum. These findings have significant implications for designing textured protein products with tailored mechanical properties. The frequency-dependent response suggests that CO is preferable for applications requiring structural stability under dynamic conditions (e.g., post 3D printing process), while SO may benefit products where paste flowability and controlled softening is desired (e.g., 3D printing extrusion process and meat analogues).

#### 2.1.3. Rheological Changes During Gel Formation of MP Paste with Different Oil Concentration

**Figure 3:**
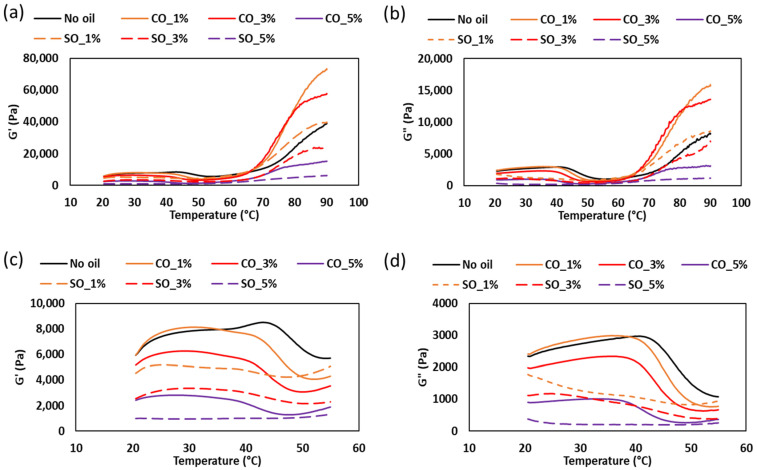
Storage modulus (G′) (**a**) and loss modulus (G″) (**b**) of MP paste during heating from 20 °C to 90 °C, showing gel formation behavior. Enlarged views of the modulus changes in the 20 °C to 55 °C range are presented in (**c**) and (**d**), respectively.

The temperature-dependent rheological behavior of MP paste during heating (20–90 °C) reveals critical transitions in protein network formation influenced by oil type and concentration. As shown in Figure 3, the G′ and G″ moduli exhibit distinct changes corresponding to lipid phase transitions, protein denaturation, and gelation processes. Paste without oil and CO_1% displayed similar G′ and G″ values until ~35 °C, suggesting minimal initial structural disruption. However, beyond this temperature, a decline in both moduli was observed for CO-containing samples, particularly around 30 °C, which coincides with the melting point of coconut oil (~24–26 °C). This reduction indicates that the solid-to-liquid transition of coconut oil temporarily weakens the protein network, likely due to increased mobility of the lipid phase within the matrix [4]. In contrast, SO, which remains liquid at room temperature, did not exhibit this sharp decline, supporting the hypothesis that lipid physical state significantly impacts early-stage gelation dynamics.

The subsequent increase in G′ and G″ after 45 °C corresponds to myosin denaturation, a critical step in MP gelation. As myosin unfolds (~45 °C), reactive groups (e.g., hydrophobic residues and sulfhydryls) become exposed, facilitating protein–protein interactions [18]. Notably, samples with low CO concentrations (1–3%) showed enhanced gel strength compared to the control, suggesting that hydrophobic interactions between lipids and unfolded proteins may stabilize the forming network. This aligns with findings by Zhou et al. [4], who reported that moderate lipid incorporation can improve gel elasticity by acting as nucleation sites for protein aggregation. At elevated temperatures (>55 °C), 1% and 3% CO samples exhibited higher G′ values than the oil-free control, indicating that CO may enhance thermal gelation by promoting hydrophobic protein aggregation. However, at 5% oil (both CO and SO), both G′ and G″ decreased, suggesting that excessive oil disrupts the continuous protein network. This phenomenon is consistent with studies by Zhou et al. [4], who observed that high lipid concentrations (>3%) introduce discontinuities in myofibrillar gels, acting as physical barriers that impede protein crosslinking. These insights highlight the importance of optimizing lipid content in surimi-based products to balance texture and structural integrity.

### 2.2. Effect of Oil Concentration on the Nonlinear Rheological Behavior of MP Paste and Gel

The effect of oil concentration on the nonlinear rheological behavior of MP was investigated in both paste and gel forms (Table 1 and Table 2). The addition of oil significantly altered the material’s structural properties, making it more prone to deformation under strain. For both paste and gel, crossover strain (i.e., the point where the material transitions from a solid-like to a fluid-like behavior) decreased with increasing oil concentration, indicating that oil weakens the protein network. In the paste, the crossover strain reduced from 119.06% (no oil) to 25.62% (SO_5%), and in the gel, it decreased from 97.54% to 48.32% for the same oil concentrations. CO showed a similar trend, with both paste and gel exhibiting reduced crossover strains as oil content increased. These results suggest that the presence of oil facilitates a quicker transition to a more liquid-like state, which is consistent with studies by Xu et al. [20], who found oil disrupted protein network formation in gel-like materials.

Critical strain (γ_c_), which represents the strain at which the material yields, also decreased with increasing oil concentration. In the paste, γ_c_ reduced from 4.05% (no oil) to 1.60% (SO_5%), and in the gel, it dropped from 6.06% to 2.23% for the same oil concentrations. These findings indicate that oil makes the material yield at lower strains, suggesting weaker structural integrity. Interestingly, in the case of CO_1%, the γ_c_ and G′ at γ_c_ values were higher in the gel state compared to the no-oil sample, but not in the paste state. Specifically, in the gel, γ_c_ increased from 6.06% (no oil) to 7.12% (CO_1%), and G′ at γ_c_ increased from 9570.77 Pa (no oil) to 9628.45 Pa (CO_1%). This suggests that the addition of 1% CO enhances the gel’s structural integrity, possibly due to favorable interactions between coconut oil and the protein network that help preserve or improve its rigidity. However, in the paste, the addition of CO_1% did not show this enhancing effect. This result aligns with studies by Zhou et al. [4], who found that small amounts of oil could improve the gelation properties of MP systems by enhancing their elasticity and network formation, but the effect may vary depending on the state of the material.

The critical storage modulus (G′_cr_), which measures the material’s resistance to deformation, also decreased significantly with increasing oil concentration in both paste and gel. For the paste, G′_cr_ dropped from 1559.12 Pa (no oil) to 994.28 Pa (SO_5%), and in the gel, it decreased from 9570.77 Pa to 745.59 Pa. This reduction in G′_cr_ reflects a weakening of the protein network due to the oil’s interference with protein–protein interactions. These findings are consistent with previous studies, including those by Zhao et al. [4], which demonstrated that oil disrupts protein networks, making the material less resistant to deformation.

Cohesive energy density (E_c_), which indicates the strength of the material’s internal structure, similarly decreased with increasing oil concentration. For both paste and gel, E_c_ values were significantly lower with higher oil content, with the paste dropping from 12.78 kJ/m^3^ (no oil) to 1.27 kJ/m^3^ (SO_5%) and the gel from 175.74 kJ/m^3^ to 1.85 kJ/m^3^. This reduction in E_c_ confirms that oil weakens the cohesive forces within the protein network especially at a concentration >3%, further supporting the notion that oil disrupts protein structure, making the material more prone to deformation.

### 2.3. Analysis of Normalized Lissajous–Bowditch Curves for MP Paste and Gel with Different Oil Concentration

**Figure 4:**
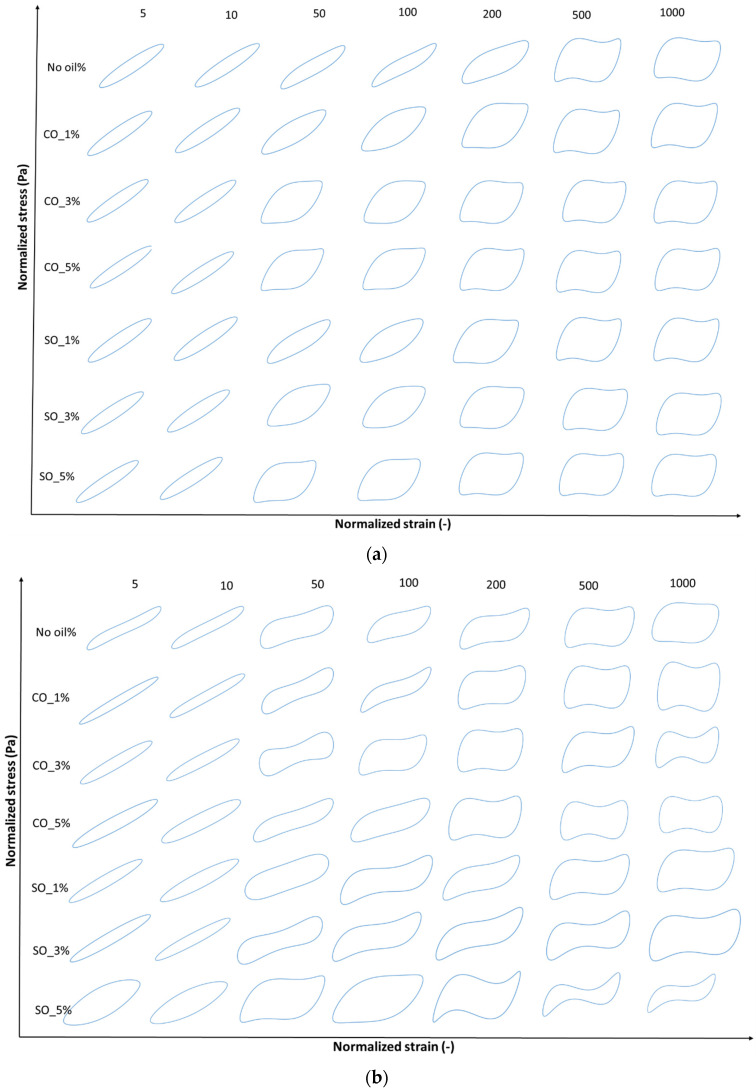
(**a**) Elastic Lissajous–Bowditch plots of MP paste at a frequency of 1 rad/s and various strain levels of 5%, 10%, 50% 100%, 200%, 500%, and 1000%. Y-axis denotes normalized shear stress (Pa) and x-axis denotes normalized shear rate (s^−1^). Strain and stress data are normalized with the maximum strain/stress in the oscillation cycle. (**b**) Elastic Lissajous–Bowditch plots of MP gel at a frequency of 1 rad/s and various strain levels of 5%, 10%, 50% 100%, 200%, 500%, and 1000%. Y-axis denotes normalized shear stress (Pa) and x-axis denotes normalized shear rate (s^−1^). Strain and stress data are normalized with the maximum strain/stress in the oscillation cycle.

**Figure 5:**
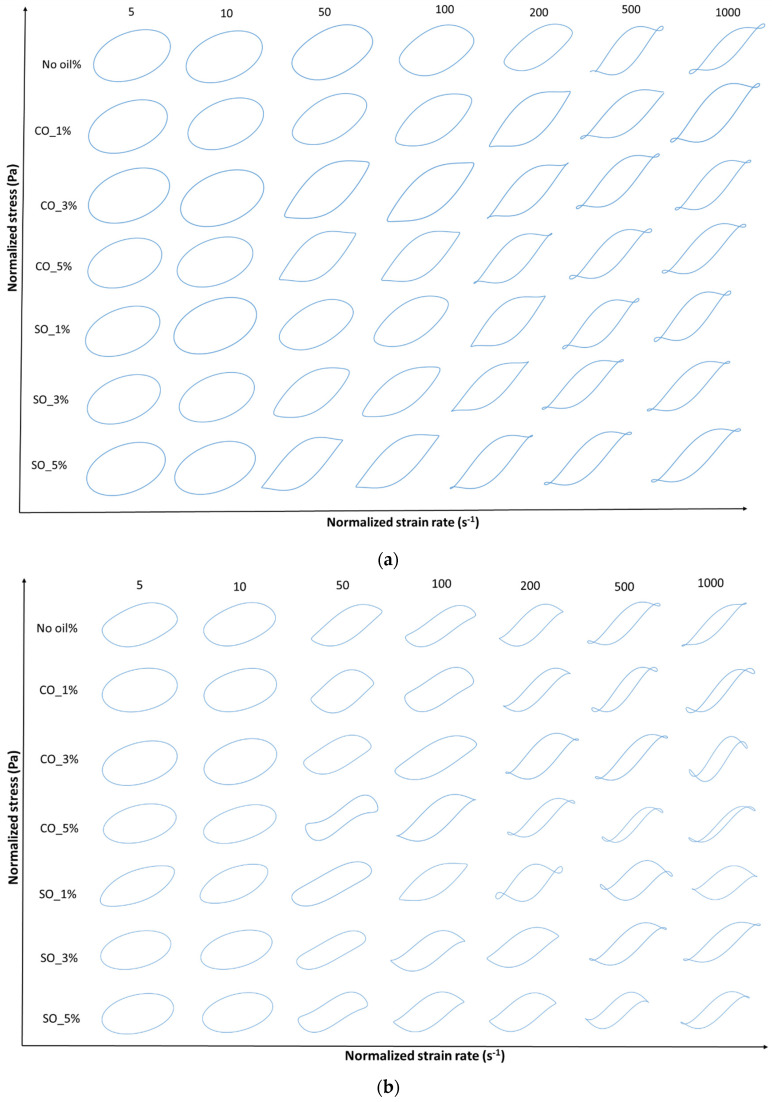
(**a**) Viscous Lissajous–Bowditch plots of MP paste at a frequency of 1 rad/s and various strain levels of 5%, 10%, 50% 100%, 200%, 500%, and 1000%. Y-axis denotes normalized shear stress (Pa) and x-axis denotes normalized strain rate (s^−1^). Strain and stress data are normalized with the maximum strain/stress in the oscillation cycle. (**b**) Viscous Lissajous–Bowditch plots of MP gel at a frequency of 1 rad/s and various strain levels of 5%, 10%, 50% 100%, 200%, 500%, and 1000%. Y-axis denotes normalized shear stress (Pa) and x-axis denotes normalized strain rate (s^−1^). Strain and stress data are normalized with the maximum strain/stress in the oscillation cycle.

LAOS tests were conducted to evaluate the nonlinear rheological behavior of MP paste and gel under large deformations. Figure 4a,b and Figure 5a,b illustrate the elastic and viscous Lissajous–Bowditch curves at 1 rad/s for strains of 5, 10, 50, 100, 200, 500, and 1000%, providing insights into the structural response of MP with different oil concentrations. These curves characterize the intracycle stress–strain relationships, revealing structural changes during deformation. These curves are particularly useful in visualizing structural rearrangements within the MP network under increasing strain, as previously demonstrated in studies on fish myofribrilar protein paste [2] and agarose–xanthan gel mixture [30].

At low strain (5%), the elastic Lissajous curves maintain an elliptical shape, indicating a predominantly linear viscoelastic response where the protein network remains intact. This is consistent with the small-amplitude oscillatory shear (SAOS) results, which showed an initial solid-like behavior in both paste and gel states. However, as the strain increases, oil-induced disruptions in the protein network become evident, leading to nonlinear stress responses. Higher oil concentrations (SO_5% and CO_5%) resulted in greater distortions in the curves, suggesting increased network instability due to weakened protein interactions. These distortions, particularly noticeable in the gel state (Figure 5b), indicate a shift towards a more fluid-like behavior at high strains, aligning with the earlier findings on crossover strain and critical strain.

At intermediate strain levels (50–100%), MP with 1% coconut oil (CO_1%) exhibits a more stable network in the gel state, as reflected in the less distorted elastic and viscous Lissajous curves compared to other oil concentrations. This suggests that at low concentrations, CO may reinforce the gel matrix, potentially through enhanced hydrophobic interactions between protein and lipid phases [4]. However, at higher oil concentrations (5%), both SO and CO disrupt the gel structure, leading to a parallelogram-like shape in the Lissajous curves, indicative of increased viscous dissipation and a transition from a gel-like to a more fluid-like behavior. This trend is more prominent in the paste, where oil-induced weakening of the protein network is more pronounced (Figure 4a).

**Figure 6:**
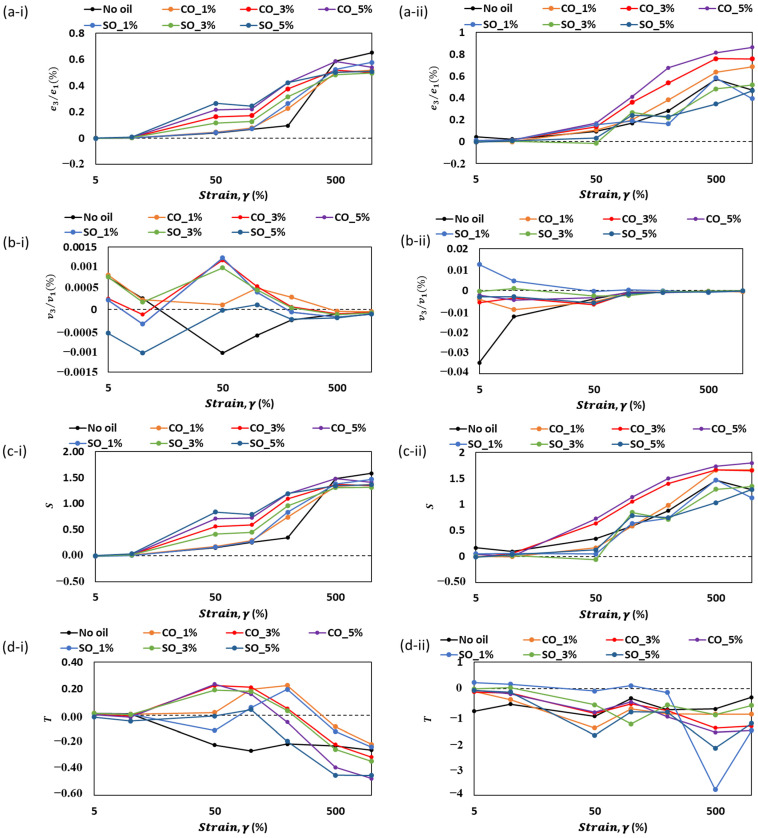
The changes in (**a**) e_3_/e_1_, (**b**) v_3_/v_1_, (**c**) S, and (**d**) T with respect to strain, γ (%), of MP paste and gel at different oil concentration. Roman numera “i” represent measurement in the paste state, while “ii” represent measurement in the gel state.

At high strain levels (200–1000%), shear-thinning behavior becomes dominant, particularly in samples with higher oil concentrations (SO_5% and CO_5%), as seen in the transition from elliptical to S-shaped Lissajous curves. The rhomboidal shapes in the viscous Lissajous plots (Figure 4b and Figure 6b) indicate that increased oil content facilitates the breakdown and rearrangement of protein aggregates, allowing the material to flow more easily. This is particularly evident in MP paste, where oil disrupts protein–protein interactions, leading to greater fluidity. These findings align with studies on oil-in-water protein emulsions, where fat incorporation reduced network rigidity and increased the nonlinearity of the stress response [31].

In summary, the Lissajous–Bowditch curves illustrate the complex interplay between oil concentration and MP structural integrity. At low concentrations (CO_1%), CO enhances the gel network, while at higher concentrations (5%), both SO and CO weaken the MP matrix, increasing nonlinear behavior and fluidity under large deformations. This knowledge is critical for optimizing MP formulations, particularly in applications where rheological stability is essential, such as in emulsion-based food products or extrusion-based processing.

### 2.4. Analysis of Chebyshev Coefficients for MP Paste and Gel with Varying Oil Type and Concentration

The Chebyshev stress decomposition method was applied to analyze the viscoelastic properties of MP paste and gel through nonlinearity indicators (e_3_/e_1_, v_3_/v_1_, S, and T). Figure 6 illustrates how these coefficients change with strain (γ) at different oil concentrations, providing insights into the influence of oil on the material’s nonlinear viscoelastic behavior. In both paste (i) and gel (ii) states, the strain-stiffening ratio (e_3_/e_1_) exhibits a general increase with strain, confirming the structural reinforcement due to protein–protein interactions (Figure 6a). The addition of oil modifies this trend, with higher oil concentrations (CO_5% and SO_5%) showing a more pronounced strain-stiffening effect, particularly in the gel state. This suggests that oil may facilitate localized interactions within the matrix, potentially enhancing structural integrity. In contrast, lower oil concentrations (CO_1% and SO_1%) display a more moderate increase, indicating a reduced influence on the network formation.

Shear thickening (v_3_/v_1_) is observed at lower strains for all samples (Figure 6b). However, the addition of oil modifies the shift from shear-thickening to shear-thinning behavior. Higher oil levels (CO_5% and SO_5%) delay this transition, maintaining positive v_3_/v_1_ values over a wider strain range, particularly in the gel state. This behavior implies that oil enhances resistance to flow at moderate strains, likely by modifying the distribution of protein interactions within the network. Conversely, samples without oil or with lower oil concentrations exhibit a quicker transition to shear thinning, suggesting weaker structural reinforcement.

The strain-stiffening ratio (S) (Figure 6c) follows a similar trend, where higher oil concentrations enhance resistance to deformation, especially at larger strains. The presence of oil likely contributes to a more cohesive network, leading to higher S values. The gel state (c-ii) exhibits more pronounced differences between oil concentrations, indicating that oil’s effect on structuring becomes more significant after gelation.

The shear-thickening ratio (T) highlights the role of oil in modulating the material’s resistance to flow (Figure 6d). In paste samples (d-i), negative T values dominate at higher strains, reflecting strain-thinning behavior across all formulations. However, the presence of oil appears to reduce the extent of this thinning, with higher oil concentrations exhibiting less negative T values. This trend is more distinct in gel samples (d-ii), where higher oil levels lead to greater variations in T, indicating that oil influences the transition from elastic to viscous response. Overall, the results suggest that oil incorporation modifies the nonlinear viscoelastic properties of MP paste and gel by influencing strain-stiffening, shear-thickening, and strain-thinning behaviors. Higher oil concentrations tend to enhance structural reinforcement, particularly in the gel state, while lower oil levels promote a more fluid-like response. These findings provide valuable insights for optimizing formulations where oil plays a crucial role in controlling rheological behavior and texture in food systems.

### 2.5. Thermal Behavior and Protein Denaturation of Oil-Incorporated MP Paste

**Figure 7:**
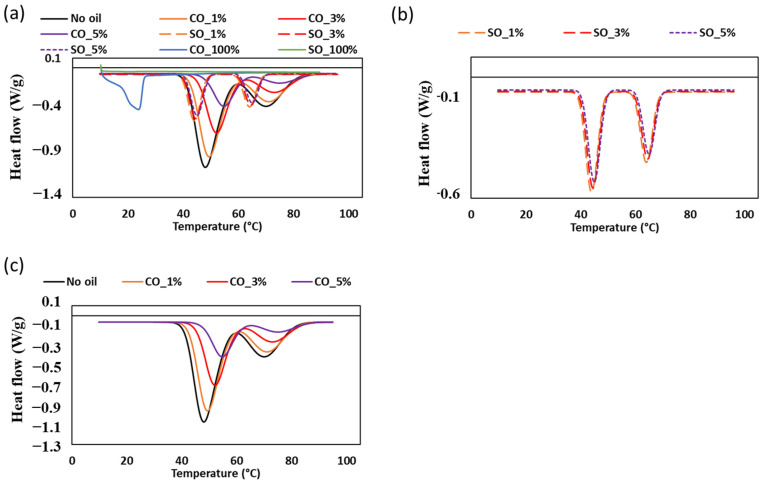
Differential scanning calorimetry thermograms of MP paste with different oil concentrations: (**a**) combined thermograms for all samples including 100% soybean and coconut oil, (**b**) samples with soybean oil, and (**c**) samples with coconut oil.

The thermal transitions of MP paste with varying concentrations of CO and SO were analyzed using differential scanning calorimetry (DSC) and illustrated in Figure 7 and Table 3. The DSC thermograms revealed two prominent endothermic peaks corresponding to the denaturation of myosin and actin, which were significantly influenced by oil type and concentration. The denaturation temperature of myosin in the control sample was approximately 48.21 °C, which increased progressively with increasing CO concentration, reaching 55.61 °C at CO_5%. Similarly, actin denaturation shifted from 68.39 °C in the control to 73.75 °C at the highest CO level. In contrast, the inclusion of SO had a minimal impact on these transition temperatures, with myosin denaturation observed within the range of 44–45 °C and actin denaturation between 64 and 65 °C.

The increase in protein denaturation temperature with CO incorporation suggests an enhanced stabilization effect. This may be attributed to the unique physical properties of CO, which is rich in saturated fats and has a higher solid fat content at room temperature [31]. The presence of solid lipid particles in CO could create a more rigid matrix within the MP paste, restricting molecular mobility and reducing water availability for protein hydration. Such interactions may lead to increased thermal stability of the protein network, consistent with previous studies demonstrating the influence of lipid structure on MP protein functionality [4]. Conversely, SO, which remains liquid at room temperature, did not induce a significant increase in denaturation temperature. This is likely due to its lower saturation level and inability to form a rigid lipid–protein matrix, allowing for more water–protein interactions that facilitate denaturation at lower temperatures. Previous studies have shown that unsaturated oils, such as SO, have a less pronounced effect on protein stability compared to saturated fats [19]. The observed differences between CO and SO treatments align with findings that suggest lipid composition plays a critical role in modifying protein thermal properties in food systems.

The enthalpy (ΔH) values derived from DSC (Table 4) further support the differential effects of CO on MP paste stability. The control sample exhibited the highest enthalpy (−20.64 J/g), while CO_5% displayed a substantial reduction (−10.45 J/g). A decrease in enthalpy indicates that less energy is required for protein unfolding, suggesting that CO disrupts the protein–protein interactions that typically contribute to the structural integrity of the MP gel [16]. In contrast, the enthalpy values of SO-containing samples showed only minor variations (−10.79 to −9.58 J/g), reinforcing the notion that SO has a less stabilizing effect on MP proteins compared to CO. Additionally, the melting of pure CO (100%) was detected at 24.78 °C, a transition that was not evident in MP paste samples (Figure 7a). This suggests that the oil was effectively dispersed within the protein matrix, leading to an indirect but significant impact on thermal behavior. The absence of a distinct melting peak in MP samples indicates that CO was integrated into the protein network rather than existing as free lipid phases, which could contribute to the observed increase in protein stability.

These findings have significant relevance to the functional properties of MP-based gels in food applications. The incorporation of CO enhances protein thermal stability, potentially improving gel firmness and water-holding capacity, which are crucial for texture and quality attributes [4]. Meanwhile, the limited impact of SO suggests that oil type must be carefully considered when designing MP formulations to achieve specific textural and stability outcomes.

### 2.6. Texture Characteristics of MP Gel with Different Concentrations of Oil

**Figure 8:**
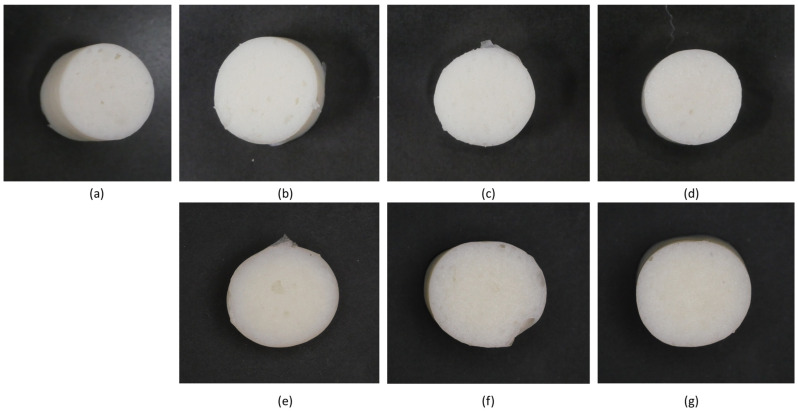
Visual appearance of MP gels containing different oil types at varying concentrations: (**a**) no oil, (**b**) CO_1%, (**c**) CO_3%, (**d**) CO_5%, (**e**) SO_1%, (**f**) SO_3%, and (**g**) SO_5%.

**Figure 9:**
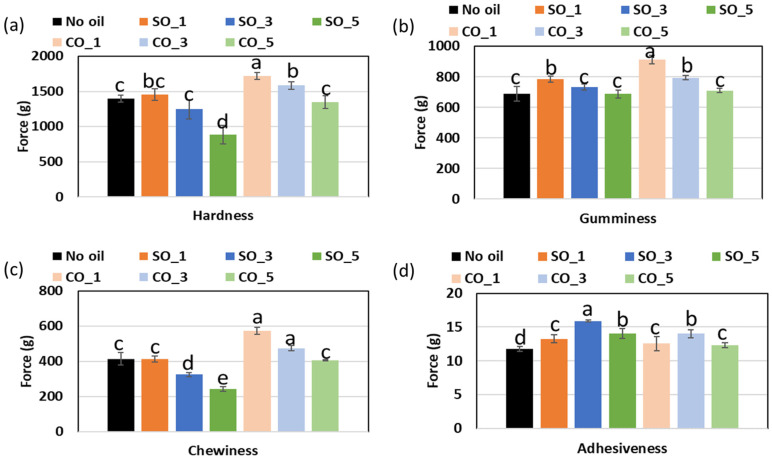
Texture characteristics of MP gel prepared with different oil concentration: (**a**) Hardness, (**b**) gumminess, (**c**) chewiness, and (**d**) adhesiveness. Barchart with different letters (a−e) significies values that are significantly different (*p* < 0.05).

The visual appearance of MP gels is shown in Figure 8, while the textural properties, including hardness, adhesiveness, gumminess, and chewiness, are shown in Figure 9. The incorporation of CO and SO influenced the texture of MP gels through their impact on protein–lipid interactions, oil entrapment within the gel matrix, and the overall stability of the protein network. Hardness, which reflects gel rigidity, was higher in CO-treated samples, particularly at CO_1% (1719.38 g) compared to the control (1396.43 g). This suggests that CO enhanced protein crosslinking, likely due to its semi-solid nature at room temperature, which allowed it to integrate into the protein matrix more effectively. Saturated lipids such as CO can interact with proteins through hydrophobic interactions, strengthening the gel network and reducing molecular mobility [4,16]. Conversely, SO-containing samples exhibited a decrease in hardness at higher concentrations (SO_5%: 886.75 g), indicating a weakening of the gel. This may be due to the inability of liquid oil to form stable interactions with the protein network, leading to phase separation and reduced gel integrity [4].

Oil entrapment within the protein matrix also played a crucial role in textural modifications. In CO-treated samples, the oil was likely immobilized within the gel structure, restricting water migration and enhancing gel firmness. The rigid lipid phase in CO could act as a filler, reinforcing the protein network and leading to increased gumminess (910.76) and chewiness (573.94) at CO_1%. Studies have shown that solid lipid particles can form microdomains within a gel, contributing to a denser and more cohesive texture [20]. On the other hand, SO, being liquid at room temperature, was more likely to remain in an unbound state within the protein network. This resulted in increased adhesiveness at SO_3% (15.89) and a weaker structure at higher concentrations, as the excess liquid oil disrupted protein–protein interactions, reducing gel strength. The differences in oil integration also influenced protein–water interactions. CO-treated gels exhibited lower adhesiveness (12.29–13.99), suggesting that the presence of solid fat reduced the availability of water molecules for binding, leading to a drier and firmer texture. In contrast, SO-treated gels had higher adhesiveness, indicating that liquid oil allowed for greater water retention, which softened the gel structure and made it more cohesive [4].

### 2.7. Effect of Oil on the Flowability and Post-Extrusion Stability of 3D-Printed MP Paste

**Figure 10:**
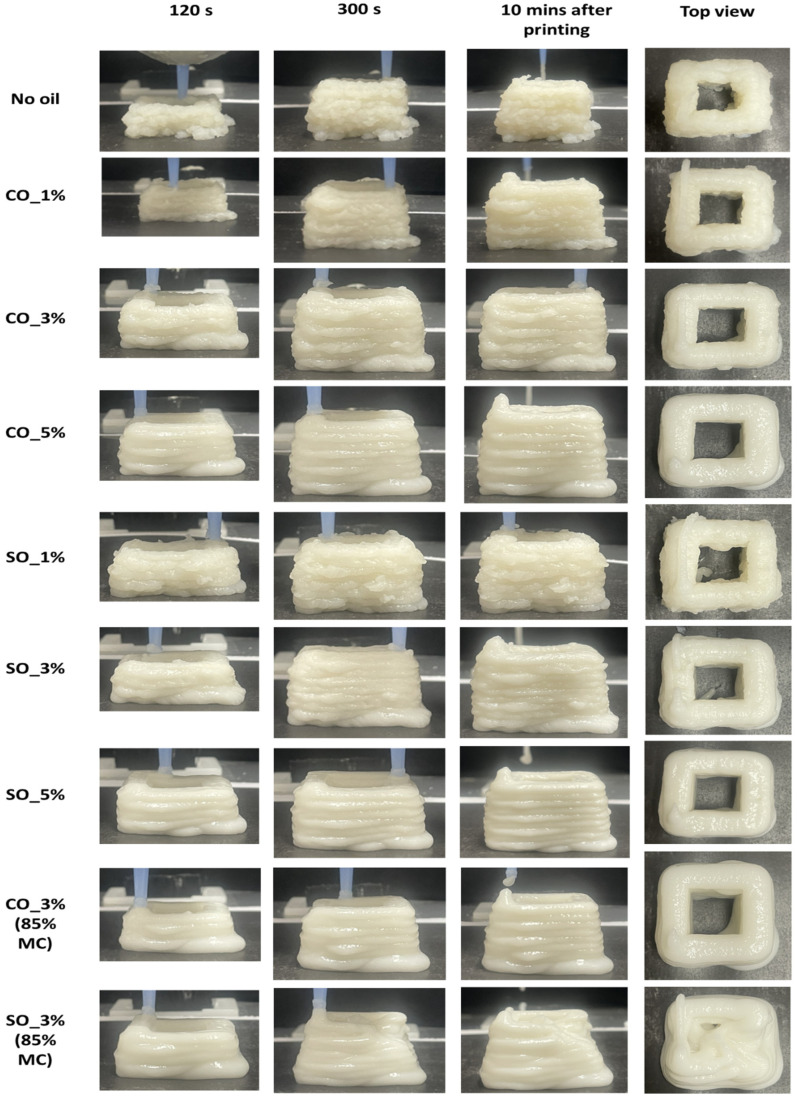
Three-dimensionally printed MP paste at different time intervals during extrusion process. The images show the paste at 120 s, 300 s, 10 min post printing and the top view of the extruded MP paste after printing.

Three-dimensional printing of MP paste was conducted to analyze the influence of oil on flowability and post-extrusion stability, both of which are crucial for maintaining extrudate structure. As shown in Figure 10, the sample without oil exhibited significant surface roughness during extrusion. This roughness likely resulted from increased pressure at the nozzle, as reported in a previous study on flow field and die swelling in 3D printing of MP paste [32]. The absence of oil may have also led to higher friction within the nozzle, causing inconsistent material deposition and exacerbating surface irregularities [33]. The addition of oil significantly reduced surface roughness, indicating improved flowability. Both CO and SO samples maintained their extrudate structure at 82% moisture content, suggesting that oil facilitated a more uniform extrusion process. Acting as a lubricant within the protein matrix, oil reduces internal friction, leading to smoother extrusion. This effect prevents abrupt ruptures and structural inconsistencies, which are common in high-deformation processes like 3D printing [33].

To further examine the effect of oil, MP paste was printed at 85% moisture content with 3% oil. CO maintained the extrudate structure, while SO resulted in deformation after seven layers were printed. The increased flowability of SO, due to its liquid state, likely caused the structure to collapse under high-moisture conditions. In contrast, CO, being semi-solid at 20 °C, provided better structural support and delayed deformation. When printing MP paste with varying oil concentrations, increasing oil levels consistently improved flowability, particularly in SO-containing samples. These samples exhibited smoother surfaces than those with CO, suggesting that SO more effectively reduced internal resistance during extrusion. However, at 85% moisture content, CO-containing samples, though structurally weaker than those at 82%, retained their shape post-extrusion. In contrast, SO-containing samples experienced deformations, reinforcing the idea that CO provides better structural stability under high-moisture conditions and large deformation.

When compared to gel-like 3D-printable materials such as alginate or gelatin-based pastes, which typically exhibit stable structures at high-moisture contents due to ionic or thermal gelation [34,35], the MP paste with CO demonstrated comparable stability without requiring external crosslinking or further cooling. This suggests that oil–protein interactions in CO-based formulations offer a promising strategy for structure retention in protein-rich pastes, especially under elevated moisture and deformation. These findings highlight the importance of oil type and concentration in optimizing printability and maintaining structural integrity in 3D printing of MP paste. The enhanced flowability and post-extrusion stability, especially in CO-containing formulations, demonstrate their potential for producing soft-textured 3D-printed products suitable for elderly individuals and people with dysphagia, where both swallowability and structural cohesion are essential.

### 2.8. Microstructure Analysis of MP Gels with Varying Oil Types and Concentrations

**Figure 11:**
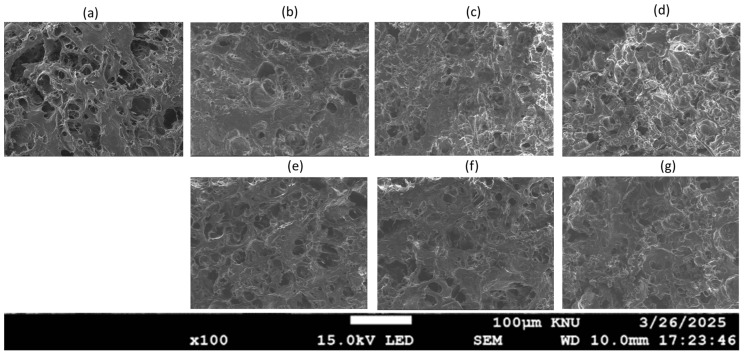
Scanning electron microscopy (SEM) images of MP gels containing different oil types at varying concentrations: (**a**) no oil, (**b**) CO_1%, (**c**) CO_3%, (**d**) CO_5%, (**e**) SO_1%, (**f**) SO_3%, and (**g**) SO_5%.

Scanning electron microscopy (SEM) was used to investigate the microstructure of MP gels containing different oils (Figure 11). The SEM images revealed distinct dispersion patterns of oil based on both type and concentration, with higher oil concentrations leading to fewer void spaces due to the oil, which could function as a filler in the protein three-dimensional network structure [4]. At 1% oil concentration, both CO and SO gels exhibited a continuous and uniform protein network, with small oil droplets dispersed throughout, indicating minimal disruption of gel structure (Figure 11b and Figure 11e, respectively). This observation aligns with findings by Zhou et al. [4], who reported that low oil concentrations (<2%) did not significantly affect the protein network. However, at 3%, differences between the oils became evident (Figure 11c,f). SO gels developed larger pores, suggesting a weakened network structure. This may be due to the weaker emulsifying effect of SO, which leads to less effective protein–oil interaction, as seen in studies by Wu et al. [6]. In contrast, CO gels remained dense with a slightly coarser, yet still interconnected network, suggesting that CO’s higher saturation level enhances fat–protein interactions and stabilizes the gel matrix [16].

At 5%, the SO gels exhibited a highly disrupted and porous structure with significant phase separation between protein and oil, indicating that excess SO interferes with protein gelation. This result is consistent with those of Wu et al. [36], who found that higher oil concentrations could hinder gel formation and disrupt the protein network. On the other hand, CO gels maintained a relatively compact structure, though larger voids were observed, suggesting partial destabilization due to high oil content. This finding supports the observations from the texture and rheological analysis of this study, highlighting that oils with higher saturation, such as CO, are more likely to preserve gel integrity even at higher concentrations.

## 3. Conclusions

This study investigated the impact of oil concentration and type on the rheological, thermal, textural, and structural properties of myofibrillar protein (MP) paste and gel, providing critical insights into their processing behavior, including 3D printability. The findings highlight the significant influence of oil incorporation on viscoelasticity, nonlinear deformation, and extrusion stability. Small-amplitude oscillatory shear (SAOS) analysis revealed that increasing oil content reduced the storage modulus (G′) and loss modulus (G″), with soybean oil (SO) exerting a stronger weakening effect on the protein network than coconut oil (CO). Frequency-dependent behavior showed that oil-modified MP pastes exhibited stronger elasticity, particularly at a lower oil concentration (<3%). Nonlinear rheological analysis (LAOS) indicated that oil addition reduced crossover strain (γ_c_), enhancing deformability with a decrease in critical strain (γ_cr_), signifying earlier structural breakdown, especially in SO-modified samples. MP gels containing <3% CO exhibited higher strain-stiffening ratios and cohesive energy density, confirming their superior network strength post-gelation. Microstructural analysis revealed that higher oil concentrations reduced void spaces, with CO promoting a more compact protein network than SO. At 5% oil concentration, the SO gels exhibited significant phase separation, while the CO gels retained a denser structure, reinforcing findings from rheological and texture analyses. Texture analysis showed that CO increased gel hardness, with CO_1% (1719.4 g) being firmer than the control (1396.4 g), whereas SO_5% significantly weakened the gel (886.8 g). Three-dimensional printing analysis revealed that SO facilitated flow properties by reducing extrusion pressure, improving material deposition during printing. However, post-extrusion stability was affected, especially at a higher moisture content (85%), where the extrudate experience model collapsed from the seventh printed later. On the other hand, CO enhanced structural stability. These findings suggest that lower concentrations of CO (<3%) in MP gels enhance gel strength and structural integrity, making them suitable for soft-textured, personalized foods intended for individuals with dysphagia or elderly populations. Moderate CO concentrations (~3%) offer a balance between printability and post-processing stability, supporting applications in 3D-printed foods where both flowability and firmness are needed. In contrast, higher CO concentrations (>3%) may be beneficial in applications where softer textures or reduced mechanical strength are desired, such as in spreadable or easily deformable protein-based products. Overall, this study provides valuable insights for tailoring MP-based formulations to meet specific textural and functional needs in targeted nutrition and advanced food-manufacturing systems.

## 4. Materials and Methods

### 4.1. Myofibrillar Protein Paste Preparation

Frozen Alaska pollock MP (FA grade, Trident Seafood, Seattle, WA, USA) with 78% moisture, 24.1% protein, 0.9% fat, and a pH of 6.8 was obtained from Pulmuone Co., Ltd. (Gangnam-gu, Seoul, Republic of Korea), and thawed at 4 °C for 12 h before further processing. The thawed MP was minced and subsequently mixed with 2% (*w*/*w*) salt to facilitate partial MP solubilization essential for the paste network formation (Table 1). Mixing was carried out at 4 °C with a universal food processor (Model UMC5, Stephan Machinery Corp., Hameln, Germany) on a low-speed setting for 1 min. The moisture content of the MP paste was adjusted to 82% by incorporating deionized water, following the method described by Oyinloye and Yoon [2]. Soybean oil (SO) and coconut oil (CO) were selected as lipid-phase components, representing oils rich in polyunsaturated and saturated fatty acids, respectively. Each oil type was incorporated into the MP paste at concentrations of 0, 1, 3, and 5 g per 100 g of paste. Oil was dispersed using a high-shear mixer operating at 5000 rpm for 5 min at 4 °C to ensure uniform emulsification throughout the mixture. The prepared MP paste samples were subsequently stored under refrigeration at 4 °C until further analysis.

### 4.2. Small-Amplitude Oscillatory Shear (SAOS) Analysis for MP Paste and Gel

The viscoelastic properties of MP paste and gels containing different oil concentrations were evaluated using SAOS measurements. A Discovery Hybrid Rheometer (Model HR-3, TA Instruments, New Castle, DE, USA) fitted with parallel plate geometry (40 mm, gap 2 mm) was used for the analysis. A strain sweep test was carried out at 20 °C, covering a strain range from 0.01% to 100% at a fixed frequency of 1 Hz, to identify the linear viscoelastic region (LVR), where the storage modulus (G′) and loss modulus (G″) remained stable with increasing strain, indicating a proportional stress–strain response. A frequency sweep test was then performed at 20 °C, spanning from 0.1 to 100 rad/s at a constant strain of 0.1%, ensuring that the measurements stayed within the LVR. To examine the thermal gelation behavior, a temperature sweep test was conducted from 20 °C to 90 °C at a heating rate of 2 °C/min. The gelation onset temperature and the impact of oil type and concentration on thermal stability were analyzed.

### 4.3. Large-Amplitude Oscillatory Shear (LAOS) Analysis for MP Paste and Gel

LAOS tests were conducted at 20 °C to evaluate the nonlinear viscoelastic properties of the MP paste and gel. A strain sweep was performed over a range of 5% to 1000% strain at a frequency of 1 rad/s. The critical strain (γ_c_), defined as the strain at which the storage modulus (G′) decreased by 5% from its initial value within the LVR, was determined. The corresponding storage modulus (G′_cr_) at γc was also obtained. The cohesive energy density (E_c_), representing the intermolecular interactions within the MP paste and gel, was calculated using the following equation [37]:

(1) $$E_{c}=\frac{1}{2}{\gamma_{c}}^{2}{G^{\prime }}_{cr},$$

where γ_c_ is the critical strain, and G′_cr_ is the corresponding storage modulus.

For further nonlinear analysis, the MITlaos program (MITlaos Ver. 2.1 Beta) was used to evaluate the Lissajous curves and Chebyshev coefficients based on the shear stress (σ) and shear strain (γ) data. The elastic (e_1_, e_3_, e_5_, …) and viscous (v_1_, v_3_, v_5_, …) Chebyshev harmonics were extracted to characterize nonlinear material behavior. The ratios e_3_/e_1_ and v_3_/v_1_ were calculated to assess elastic and viscous nonlinearity, respectively. A positive e_3_/e_1_ ratio indicated strain stiffening, while a negative value suggested strain thinning. Similarly, a positive v_3_/v_1_ ratio represented shear thickening, whereas a negative value indicated shear thinning. Additionally, nonlinear viscoelastic properties were described using the following equations [38,39]:

(2) $${G^{\prime }}_{M}={\left.\frac{d\sigma }{d\gamma }\right|}_{\gamma =0}\approx e_{1}-{3e}_{3}+{5e}_{5}-{7e}_{7}+\cdots ,$$

(3) $${G^{\prime }}_{L}={\left.\frac{\sigma }{\gamma }\right|}_{\gamma =\pm \gamma_{0}}\approx e_{1}+e_{3}+e_{5}+e_{7}+\cdots ,$$

where e_1_, e_3_, e_5_, … are the elastic Chebyshev coefficients. Here, G′_M_ represents the modulus at zero strain (maximum shear rate), while G′_L_ corresponds to the modulus at the maximum applied strain (γ_0_). The nonlinear viscous properties were evaluated using:

(4) $${\eta^{\prime }}_{M}={\left.\frac{d\sigma }{d\dot{\gamma }}\right|}_{\dot{\gamma }=0}\approx v_{1}-{3v}_{3}+{5v}_{5}-{7v}_{7}+\cdots ,$$

(5) $${\eta^{\prime }}_{L}={\left.\frac{\sigma }{\dot{\gamma }}\right|}_{\dot{\gamma }=\pm {\dot{\gamma }}_{0}}\approx v_{1}+v_{3}+v_{5}+v_{7}+\cdots ,$$

where ${\eta^{\prime }}_{M}$ and ${\eta^{\prime }}_{L}$ denote the apparent viscosities at zero and maximum shear rate (γ), respectively, and v_1_, v_3_, v_5_, … are the viscous Chebyshev coefficients.

Two additional parameters, the stiffening ratio (S) and thickening ratio (T), were used to quantify nonlinear behavior:

(6) $$S=\frac{{G^{\prime }}_{L}-{G^{\prime }}_{M}}{{G^{\prime }}_{L}}\approx \frac{{4e}_{3}-{4e}_{5}+{8e}_{7}+\cdots }{e_{1}+e_{3}+e_{5}+e_{7}+\cdots }$$

(7) $$T=\frac{{\eta^{\prime }}_{L}-{\eta^{\prime }}_{M}}{{\eta^{\prime }}_{L}}\approx \frac{{4v}_{3}-{4v}_{5}+{8v}_{7}+\cdots }{v_{1}+v_{3}+v_{5}+v_{7}+\cdots }$$

Positive S values indicate strain stiffening, while negative values suggest strain thinning. Likewise, a positive T value represents intracycle shear thickening, whereas a negative value indicates shear thinning [9].

### 4.4. Thermal Analysis for MP Paste

The thermal characteristics of MP paste were evaluated using a Discovery Series differential scanning calorimeter (DSC) (TA Instruments, New Castle, DE, USA). Approximately 10–15 mg of each sample was accurately weighed (±0.01 mg) and sealed in a stainless-steel DSC pan. An empty sealed pan was used as the reference. The samples were subjected to a controlled heating process from 20 °C to 95 °C at a rate of 2 °C/min within a DSC cell, which was continuously purged with nitrogen gas flowing at 50 mL/min. The denaturation temperature (Td) and enthalpy of denaturation (ΔH) were determined from the thermograms by analyzing the peak transition temperature and endothermic peaks using Trios software (v5.0.0, TA Instruments, New Castle, DE, USA). The enthalpy values were expressed in J/g protein, and all measurements were conducted in triplicate.

### 4.5. Texture Properties

The MP paste was transferred into cylindrical protein casings (20 mm in diameter, 100 mm in length) and subjected to thermal treatment at 90 °C for 30 min in a water bath to promote gel formation. Following heating, the gels were immediately cooled in ice water and stored at 4 °C for 12 h to stabilize their structure before further analysis. The texture properties of the gels were assessed with a texture analyzer (TA.XT Plus, Stable Micro Systems, Surrey, UK). Prior to testing, the samples were equilibrated at room temperature (~25 °C) for 30 min. A spherical probe (25 mm in diameter) was used to compress the gel sample (15 mm in height). The compression was performed at a constant speed of 1 mm/s, with additional test parameters set as follows: pre-test speed of 2.00 mm/s, post-test speed of 10.00 mm/s, trigger force of 5.0 g, and a target strain of 40% [40].

### 4.6. 3D Printing Analysis

**Figure 12:**
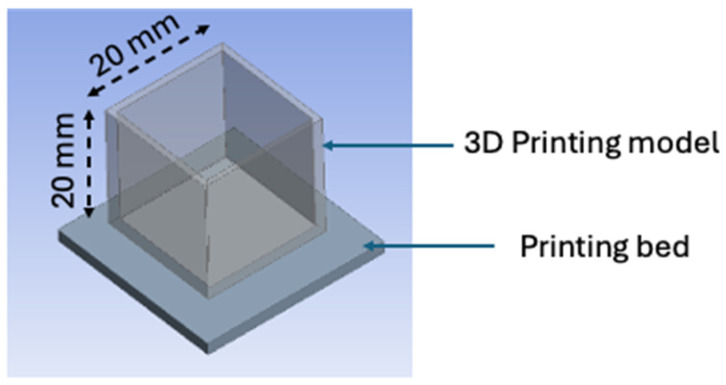
Schematic representation of the 3D-printed model for MP paste.

The extrusion-based 3D printing of MP paste containing different oil concentrations was carried out using a syringe-based fused deposition modeling (SFDM) printer (SHINNOVE-S2, Shinnove Co., Ltd., Hangzhou, China) within a temperature-controlled chamber set at 20 °C. The printing parameters, including a nozzle diameter of 1.00 mm, a layer height of 1 mm, and a travel speed of 1.5 mm/s, were selected based on previous studies to optimize deposition accuracy and structural integrity [32]. To evaluate the flowability and shape retention of the MP paste during printing, an open-box structure (20 mm × 20 mm × 20 mm) was printed (Figure 12). Extrusion consistency and filament formation were observed in real time, while the structural stability of the printed construct was assessed by monitoring shape fidelity and dimensional accuracy post-printing. Changes in the geometry and surface characteristics of the extruded material were documented using a high-resolution digital camera (Model DSLR-500D, Canon Inc., Tokyo, Japan) throughout the printing process.

### 4.7. Microstructural Characterization of MP Gel

The surface morphology of the MP gel was examined with a variable pressure field emission scanning electron microscope (VP-FE-SEM) (Model SUPRA 55VP, Carl Zeiss, Oberkochen, Germany), following a modified procedure based on the work of Yu et al. [41]. Gel samples were cut into slices with a thickness of 2–3 mm and pre-frozen at −25 °C for 12 h. The frozen samples were then subjected to vacuum freeze-drying at −50 °C for 36 h using a lyophilizer (Christ Alpha 1–4, Martin Christ, Osterode am Harz, Germany) to remove residual moisture. Once dried, the samples were mounted onto a copper grid and sputter-coated with a thin layer of gold via sputtering to enhance conductivity. SEM imaging was conducted at an accelerating voltage of 15 kV and a working distance of 10.00 mm. Micrographs were captured at 100× magnification to allow for the visualization of the overall network structure and oil dispersion within the protein gel matrix. A scale bar of 100 µm was included in the micrographs to facilitate interpretation of the void and droplet sizes within the gel structure.

## Figures and Tables

**Table 1:** The cross over strain (%), critical strain $\left(\gamma_{c}\right)$, the corresponding storage modulus $\left({G^{\prime }}_{cr}\right)$ at critical strain, and the cohesive energy density of MP paste at different oil contents.

Oil Content (%)	Crossover Strain (%)	$\mathrm{Critical}\;\mathrm{Strain},\;\gamma_{c}$ (%)	$G^{\prime }\;\mathrm{at}\;\mathrm{Critical}\;\mathrm{Strain},\;{G^{\prime }}_{cr}$ (Pa)	$\mathrm{Cohesive}\;\mathrm{Energy}\;\mathrm{Density},\;E_{c}$ (kJ/m^3^)
No oil	119.06 ± 1.36 ^a^	4.05 ± 0.23 ^a^	1559.12 ± 19 ^b^	12.78 ± 0.002 ^b^
SO_1%	44.61 ± 1.47 ^c^	3.12 ± 0.67 ^c^	1341.76 ± 21 ^d^	6.53 ± 0.002 ^d^
SO_3%	27.17 ± 1.12 ^e^	2.81 ± 0.51 ^d^	969.86 ± 17 ^f^	3.82 ± 0.001 ^e^
SO_5%	25.62 ± 1.22 ^e^	1.60 ± 0.22 ^f^	994.28 ± 23 ^f^	1.27 ± 0.001 ^f^
CO_1%	48.99 ± 1.12 ^b^	3.87 ± 0.31 ^b^	1793.46 ± 22 ^a^	13.43 ± 0.002 ^a^
CO_3%	41.12 ± 0.97 ^d^	3.04 ± 0.42 ^c^	1467.83 ± 25 ^c^	6.78 ± 0.001 ^c^
CO_5%	38.56 ± 1.02 ^d^	2.56 ± 0.47 ^e^	1063.32 ± 23 ^e^	3.81 ± 0.001 ^e^

Values in the same column marked with different superscripts (^a−f^) are significantly different (*p* < 0.05).

**Table 2:** The cross over strain (%), critical strain $\left(\gamma_{c}\right)$, the corresponding storage modulus $\left({G^{\prime }}_{cr}\right)$ at critical strain, and the cohesive energy density of MP gel at different oil contents.

Oil Content (%)	Crossover Strain (%)	$\mathrm{Critical}\;\mathrm{Strain},\;\gamma_{c}$ (%)	$G^{\prime }\;\mathrm{at}\;\mathrm{Critical}\;\mathrm{Strain},\;{G^{\prime }}_{cr}$ (Pa)	$\mathrm{Cohesive}\;\mathrm{Energy}\;\mathrm{Density},\;E_{c}$ (kJ/m^3^)
No oil	97.54 ± 1.36 ^a^	6.06 ± 0.23 ^a^	9570.77 ± 19 ^b^	175.74 ± 0.022 ^b^
SO_1%	86.15 ± 1.47 ^c^	6.11 ± 0.67 ^c^	5833.92 ± 21 ^d^	108.89 ± 0.018 ^d^
SO_3%	72.37 ± 1.12 ^e^	4.87 ± 0.51 ^d^	3713.41 ± 17 ^f^	44.04 ± 0.006 ^e^
SO_5%	48.32 ± 1.22 ^e^	2.23 ± 0.22 ^f^	745.59 ± 23 ^f^	1.85 ± 0.011 ^f^
CO_1%	103.45 ± 1.12 ^b^	7.12 ± 0.31 ^b^	9628.45 ± 22 ^a^	244.05 ± 0.032 ^a^
CO_3%	101.36 ± 0.97 ^d^	5.42 ± 0.42 ^c^	5088.63 ± 25 ^c^	74.74 ± 0.001 ^c^
CO_5%	91.63 ± 1.02 ^d^	3.69 ± 0.47 ^e^	1064.38 ± 23 ^e^	7.25 ± 0.007 ^e^

Values in the same column marked with different superscripts (^a−f^) are significantly different (*p* < 0.05).

**Table 3:** Thermal transition properties of MP paste with varying coconut oil (CO) and soybean oil (SO) concentrations.

Oil Content (%)	Peak Melting Temp (°C)	Gelation Onset (°C)	First Peak Temperature (°C)—Myosin Denaturation	Second Peak Temperature (°C)—Actin Denaturation	Enthalpy [ΔH] (J/g)
No oil	-	38.34	48.21	68.39	−20.64
CO_1%	-	40.82	49.69	69.02	−18.60
CO_3%	-	44.27	52.15	71.33	−14.52
CO_5%	-	45.75	55.61	73.75	−10.45
SO_1%	-	39.84	44.76	64.46	−10.79
SO_3%	-	39.91	44.92	64.72	−10.47
SO_5%	-	40.35	45.26	65.45	−9.58
100% soybean oil	-	-	-	-	−0.43
100% coconut oil	24.78	-	-	-	−3.18

The ‘-’ symbol indicates that the corresponding value was either not detected, not applicable, or could not be determined under the experimental conditions.

**Table 4:** Material composition for MP paste with oil.

	No Oil	Coconut Oil			Soybean Oil		
Myofibrillar protein (g)	74.45	69.78	60.45	51.07	69.78	60.45	51.07
Salt (g)	2	2	2	2	2	2	2
Ice (g)	23.55	27.22	34.55	41.93	27.22	34.55	41.93
Oil (g)	0	1	3	5	1	3	5
Total (g)	100	100	100	100	100	100	100
Sample code	No oil	CO_1%	CO_3%	CO_5%	SO_1%	SO_3%	SO_5%

CO refers to coconut oil, while SO refers to soybean oil in MP. The numerical values indicate the oil concentration levels.

## Data Availability

The data presented in this study are available in this article. Further inquiries can be directed to the corresponding author.
